# How Does Psychosocial Behavior Contribute to Cognitive Health in Old Age?

**DOI:** 10.3390/brainsci7060056

**Published:** 2017-05-23

**Authors:** Robert S. Wilson, David A. Bennett

**Affiliations:** 1 Rush Alzheimer’s Disease Center, Rush University Medical Center, Chicago, IL 60612, USA; david_a_bennett@rush.edu; 2 Department of Neurological Sciences, Rush University Medical Center, Chicago, IL 60612, USA; 3 Department of Behavioral Sciences, Rush University Medical Center, Chicago, IL 60612, USA

**Keywords:** neuroticism, loneliness, well-being, mild cognitive impairment, dementia, longitudinal study, clinical-pathologic study, neuropathologic examination

## Abstract

With the aging of the U.S. population, the number of cognitively disabled persons is expected to substantially increase in coming decades, underscoring the urgent need for effective interventions. Here, we review the current evidence linking psychosocial factors to late-life cognitive loss and consider the study design needed to illuminate the biologic bases of the associations. We then examine an ongoing study that includes several of the key design elements, the Rush Memory and Aging Project. In this longitudinal clinical-pathological cohort study, indicators of personality, social connectedness, and psychological well-being were shown to predict late-life cognitive outcomes. Participants who died underwent a uniform neuropathologic examination to quantify common dementia-related pathologies. Some psychosocial indicators were associated with cerebral infarction; some indicators modified the association of neurodegenerative pathologies with cognitive loss; and the association of some indicators with cognitive outcomes appears to be independent of the pathologies traditionally associated with late-life dementia. These findings suggest that psychosocial behavior influences late-life cognitive health through multiple neurobiologic mechanisms. A better understanding of these mechanisms may lead to novel strategies for preserving cognitive health in old age.

## 1. Introduction

The age of the population of the United States and much of the rest of the world is projected to increase in the coming decades. Without effective interventions, it is therefore likely that age-related conditions will be more common. Dementia, which is characterized by progressive cognitive decline and impairment in multiple cognitive domains [1,2], is robustly related to age. Although dementia is known to occur before the age of 65, it is relatively rare. By contrast, among persons aged 85 years or older, the fastest growing age group in the population, approximately 40% are estimated to meet dementia criteria [3]. Because of this demographic shift, the number of people with dementia in the United States is expected to double or triple by mid-century [3], representing a substantial challenge to our health care system, even if the overall prevalence of late-life dementia is slightly decreasing, as suggested by some epidemiologic data from the United States [4,5] and Europe [6]. These observations underscore the urgent need for interventions to help maintain cognitive functioning in old age.

Most dementia reflects a mix of pathologic changes in the brain [7,8,9,10,11]. Those most commonly seen during a postmortem examination of the brains of older persons include neuritic plaques, neurofibrillary tangles, Lewy bodies, transactive response DNA-binding protein 43 (TDP-43) pathology, hippocampal sclerosis, cerebral infarcts, atherosclerosis, arteriolosclerosis, and cerebral amyloid angiopathy. Dementia occurs toward the end of chronic disease processes that can be divided into asymptomatic and symptomatic phases [12]. The symptomatic phase is thought to consist of gradually accelerating cognitive decline and other subtle changes in behavior that precede the point at which dementia criteria are met by approximately one [13,14] or two [15] decades. The asymptomatic phase is thought to consist of the accumulation of neurodegenerative and vascular changes in the brain over many years. Precursors of Alzheimer’s disease (AD), for example, have been identified during a postmortem examination of the brains of young adults [16].

Research on cognition in old age has traditionally treated cognitive change within the normal range as entirely distinct from cognitive change in the impaired range (i.e., MCI and dementia). However, the long periods of cognitive decline preceding diagnoses of MCI and dementia [13,14,15] underscore how difficult it is to separate the incipient stages of these conditions from normal cognitive functioning. In addition, the neurodegenerative and cerebrovascular pathologies traditionally associated with MCI and dementia have been shown to be robustly related to incipient mild changes in cognition [17,18,19]. Therefore, on the basis of current evidence, we think it is more parsimonious to assume that the mechanisms underlying late life cognitive loss are broadly similar across the spectrum of cognitive ability.

Although these age-related pathologic changes in the brain are thought to be the main drivers of late-life dementia and cognitive decline, it has long been recognized that the correlation of these pathologies with variability in late-life cognitive functioning is imperfect [20]. For example, recent studies suggest that dementia-related pathologies account for less than half of the variance in late-life cognitive decline [18,19]. These modest cognitive-pathologic correlations suggest that factors other than dementia-related pathologies are contributing to the risk of late-life dementia.

A wide range of demographic, genetic, medical, and experiential measures have been associated with the risk of dementia. The focus of this article is on the psychosocial aspects of life experience, including personality traits, psychological traits, and social connectedness. Specifically, we will examine the current knowledge on the association of psychosocial behaviors with late-life cognitive health, consider how advances in study design and operations might address gaps in current knowledge, and briefly describe findings from an ongoing longitudinal clinical-pathologic cohort study that includes several of these design and operational components, the Rush Memory and Aging Project [21,22].

## 2. Psychosocial Factors and Cognition

### 2.1. Big Five Personality Traits

In the past 15 years, observational studies have examined associations between the big five personality traits and cognition in old age. Perhaps the most investigated trait has been neuroticism. Individuals differ in their proneness to experience psychological distress (in the form of negative emotions such as depression and anxiety). This trait, variously referred to as neuroticism [23], emotional instability [24], and negative affectivity [25], is included in most typologies of personality. Although some change in the trait has been observed during adulthood, possibly in response to negative life events [26,27], it is generally quite stable, with six-year test-retest correlations approaching unity in one study [28]. Therefore, the level of the neuroticism trait in old age provides an indicator of the cumulative level of psychological stress experienced across the life span. A higher level of neuroticism has been associated with adverse cognitive outcomes, including an increased risk of mild cognitive impairment (MCI) [29] and dementia [30,31,32,33,34], and a more rapid rate of cognitive decline [35,36,37,38,39]. Some studies have not observed an association of neuroticism with cognitive outcomes [40,41], perhaps reflecting methodological factors [42], but also suggesting that the magnitude of the association may be modest.

The neuroticism trait is robustly associated with the future occurrence of negative emotions such as depression [43]. Many studies have examined the relation of depressive symptoms to subsequent cognitive outcomes, with most results supporting an association with the risk of dementia. Thus, a higher level of depressive symptoms has been associated with a higher incidence of dementia [44,45,46,47] and MCI [48,49], and a more rapid rate of cognitive decline [44,50,51], with dual change models suggesting that the association is mainly due to depressive symptoms predicting subsequent changes in cognition [52]. In addition, individuals with a clinical diagnosis of depression are more likely to develop dementia [53]. Although negative results have been reported, these have often been contradicted by later studies of the same cohort or are attributable to methodological shortcomings [42].

Psychological distress can arise from exposure to stressors. However, the association between exposure to psychosocial stressors and late-life cognitive health has not been extensively investigated and results have been inconsistent. The strongest evidence for an association comes from a Scandinavian study that assessed psychological stress with a single question. A higher level of stress at midlife predicted a higher risk of dementia in old age after controlling for chronic psychological distress [54]. A link between negative life events and subsequent cognitive outcomes has been observed in some studies [55,56], but other studies have found mixed [57] or no [58,59] evidence of an association.

Another approach has been to quantify psychological stress in the workplace. An initial study found that higher work-related stress, as indicated by low job control and high job demands, was associated with a higher risk of late-life dementia [60]. However, support for this finding has been mixed in subsequent research [61,62].

In animal models, chronic stress is associated with functional and structural changes in the brain, particularly the limbic-hypothalamic-pituitary-adrenal axis [63]. The hippocampal formation and hippocampally mediated forms of learning and memory appear especially vulnerable [64]. Thus, chronic psychological distress may gradually compromise memory systems so that relatively less dementia-related pathology is needed to impair function in old age. In this regard, it is noteworthy that chronic anxiety and depression have been associated with decreased dendritic arborization in the CA3 subfield of the hippocampus [65], a prominent finding in animals exposed to chronic stress [64,65].

There has been less research on the other four of the big five personality traits. A higher level of conscientiousness, which refers to goal directedness and self-control, has been consistently associated with slower cognitive decline [39,66,67] and a reduced risk of developing cognitive impairment [34] and dementia [32,34,68]. Openness to experience denotes intellectual curiosity and interest in learning. A higher level of the trait has been associated with a higher level of cognition [66] and reduced cognitive decline [39,69]. Meta-analyses suggest a marginal association between openness and risk of dementia [32,68]. Higher levels of extraversion [39] and agreeableness [39,68] have been linked to better cognitive outcomes in some studies, but these associations have not been consistently observed.

### 2.2. Social Connectedness

Much human activity is devoted to social connections, and the brain is the “central organ for forming, monitoring, maintaining, repairing, and replacing salutary connections with others” [70]. It is not unreasonable to suppose, therefore, that different levels of social connectedness in old age might be associated with cognitive health, and epidemiologic research has provided some support for this idea.

Several studies have asked persons to rate how often they engage in common social activities, such as volunteering in community activities or visiting friends or relatives. In most [71,72,73,74,75], but not all [76] of these studies, more frequent participation in late-life social activities has been associated with better cognitive outcomes. The results for social network size have been mixed, with a larger network size related to better cognitive outcomes in some studies [77,78], but not others [79,80].

Some of this inconsistency may reflect variation in the quality, rather than the quantity, of social interactions. Thus in one study, it was not the size of social networks that predicted the subsequent risk of dementia, but how satisfying and reciprocal they were [80]. Along similar lines, more frequent negative social interactions, such as the unsolicited criticism of an individual or excluding an individual from a group activity, have been associated with adverse health outcomes such as depression [81,82,83] and disability [84,85]. The relation of negative social interactions to late-life cognitive health has not been extensively investigated, but one study found more frequent negative social interaction to be related to an increased risk of developing MCI [59]. This association appeared to be mainly due to the correlation of negative social interactions with a lower level of cognition, rather than with a faster rate of cognitive decline. It also may be that satisfying interactions tend to be cognitively stimulating and thereby benefit cognition [42].

Some of the inconsistent findings with objective measures of social connectedness may be because they do not adequately capture the emotional dimension of connectedness. Individuals differ in how connected they feel to others and this is only weakly related to more objective indicators of social engagement. People who feel less connected to others (i.e., lonelier) experience more rapid cognitive decline [86,87,88,89] and are more likely to develop dementia [90,91] than persons who feel more connected, even after adjusting for objective indicators of social connectedness and depressive symptoms.

The neurobiologic basis of the association of loneliness with cognition in old age is not well understood. Several factors associated with late-life loss of cognition, including an elevated blood pressure, low physical activity, depressive symptoms, and sleep fragmentation, are also related to loneliness [92,93,94,95]. Therefore, it may be that loneliness partly mediates these associations. Loneliness has also been associated with neuroradiologic evidence of cortical amyloid burden [96], a neuropathologic sign of AD, suggesting that loneliness might also be related to cognition through an association with dementia-related pathology. In addition, loneliness is associated with alterations in gene expression, including the increased activity of pro-inflammatory genes [97] that may compromise brain function.

### 2.3. Well-Being

Well-being is widely viewed as an essential component of healthy aging. Consistent with this idea, a higher level of well-being has been associated with reduced morbidity [98,99,100,101] and mortality [102] in longitudinal studies. However, the association of well-being with late-life cognitive health has not been extensively studied.

An important early prospective study of well-being and cognition was based on 13 years of data collected in the Berlin Aging Study [103]. In a dual change score model, a higher level of well-being predicted less subsequent decline in perceptual speed. Further, the level of perceptual speed did not predict the subsequent change in well-being, consistent with a previous longitudinal study [104]. However, a more recent study found evidence of a bidirectional relationship between well-being and cognition [105].

These observations suggest that a higher level of well-being in old age is associated with better cognitive health. However, the basis of the association is uncertain. It is possible that lower well-being is partly a consequence of cognitive loss or that well-being somehow modifies the impact of age-related brain pathologies on cognition. For example, positive emotions are hypothesized to broaden thought-action repertoires; thereby building intellectual, social, and psychological resources [106] and perhaps cognitive reserve. Alternatively, the mechanisms linking well-being with cognition may be independent of common dementia-related pathologies. In addition, well-being is not a unitary construct [107], and it is possible that dimensions of well-being are differentially related to late-life cognitive functioning.

### 2.4. Methodologic Challenges

Any attempt to understand how a given psychosocial factor impacts late-life cognitive functioning must confront substantial challenges. First, because it appears that incipient cognitive and neuropathologic changes begin to appear prior to old age, a longitudinal study should enroll individuals early in the life span, before these changes begin to occur. That is, to the extent that late-life cognition depends on childhood experiences (e.g., early life cognitive ability [108], cognitive activity [109], and socioeconomic status [110,111]), the ideal longitudinal study would need to enroll individuals early in the life span to account for all potential mechanisms and covariates.

A second challenge is that because the dementia syndrome has such a chronic course with the onset of its asymptomatic and symptomatic phases difficult to identify, it can be hard to distinguish a risk factor from a prodromal manifestation of dementia. This is particularly true for potentially modifiable behavioral risk variables that are possible targets of interventions, such as psychosocial behaviors. The most definitive way to confront this issue is to conduct a uniform postmortem examination of the brain to quantify dementia-related pathologies. If one or more of these pathologies are related to the behavior in question, it is a potential disease manifestation. If the pathologic markers are not related to the behavior in question, it is likely to be a risk factor. This approach is particularly important when participants in a cognitive aging study are enrolled late in the life span.

Another challenge in research on cognitive reserve and dementia is that, as noted above, dementia and MCI are usually the result of multiple pathologic processes, several of which cannot currently be reliably identified without a detailed neuropathologic examination (e.g., microinfacts, Lewy bodies, TDP-43). Thus, to quantify the full impact of these pathologies on cognition, a vital first step in assessing cognitive reserve, a postmortem neuropathologic examination is of crucial importance. Such an examination also allows for the possibility that cognitive reserve effects may vary across different pathologic processes.

Another challenge in research on late-life cognition is accounting for the impact of impending death. Much of late-life cognitive decline occurs in the last few years of life, with relatively little cognitive loss preceding this terminal period [112,113,114,115]. Thus, it is difficult to model late-life cognitive changes without knowing the time until death. Although dementia-related pathologies are associated with both preterminal and terminal cognitive decline [17,18,116], a substantial portion of terminal decline is not associated with these pathologies. It is quite possible, therefore, that cognitive reserve may differentially relate to preterminal versus terminal cognitive change [117].

In summary, research on how psychosocial factors hasten or delay the development of late-life dementia faces many challenges. The ideal study would enroll participants early in life; collect longitudinal data on (psychosocial, genomic, and medical) risk factors, cognition, and biomarkers until death; and conduct a uniform postmortem examination of the brain to quantify pathologies traditionally associated with dementia and putative markers of neural reserve. To date, we are not aware of scientific results from a study that includes all of these components, but there are studies that incorporate some of these components. We will describe one such study, the Rush Memory and Aging Project, and its findings to date on the psychosocial components of cognitive aging.

## 3. Rush Memory and Aging Project

### 3.1. Study Design and Operations

The Rush Memory and Aging Project is an ongoing longitudinal clinical-pathologic cohort study that began in 1997 [21,22]. Eligibility is determined at the time of enrollment. Inclusion criteria are age >50, agreement to annual clinical evaluations, and a brain autopsy and neuropathological examination upon death. The only exclusion criterion is a prior clinical diagnosis of dementia. Individuals are recruited from retirement communities, subsidized housing facilities, social service agencies, and churches. After a presentation about the project, interested individuals discuss it further with study staff, who obtain written informed consent and an Anatomic Gift act for the donation of the brain, spinal cord, nerves, and muscle. The project was approved by the institutional review board of Rush University Medical Center.

At the time of enrollment and annually thereafter, participants undergo a uniform clinical evaluation that includes an assessment of the possible risk factors, structured medical history, neurological examination, and detailed cognitive and motor testing. On the basis of this evaluation, an experienced clinician diagnoses MCI, dementia, AD, and other common chronic conditions of old age. Clinical classification at each annual evaluation is done blinded to all previously collected data. As a result, incident diagnoses (i.e., MCI, dementia) and the change in cognitive function are relatively independent outcomes, and analyses of cognitive change can be used to validate the clinical classification system [118].

At death, the brain is removed and examiners, blinded to all clinical data, follow a standard protocol for preserving and sectioning the tissue and quantifying the pathologic findings [119,120]. Slabs are visually examined for gross infarcts. Nine regions in one hemisphere are examined for microscopic infarcts with 6-μm sections embedded in parafin and stained with hematoxilyn and eosin [121]. Cerebral atherosclerosis is rated on a six-point scale based on a gross inspection of the anterior, middle, and posterior cerebral arteries at the circle of Willis; cerebral arteriolarsclerosis is rated on a six-point scale based on a microscopic examination of sections from the anterior basal ganglia [122]. Cerebral amyloid angiopathy is based on ratings of amyloid deposition on meningeal and parenchymal vessels from four neocortical regions [123].

Beta-amyloid plaques and tau-tangles are assessed in eight brain regions using immunohistochemistry and computer-assisted sampling, with regional scores averaged to yield composite measures [124]. Lewy bodies are identified in six brain regions using monoclonal antibodies to alpha-synuclein [125]. Severe neuronal loss in the pyramidal cell layer of the subiculum or any hippocampal subfield is classified as hippocampal sclerosis [126]. TDP-43 pathology is assessed in six brain regions using monoclonal antibodies to phosphorylated TDP-43 [127] that stains pathologically phosphorylated TDP-43, but not normal nuclear TDP-43 [126,128].

Enrollment in the Rush Memory and Aging Project is ongoing. As of 1 March 2017, 1895 individuals had enrolled and completed the initial clinical evaluation (mean age at baseline = 80.0, SD = 7.6, range: 60–99). A total of 1664 persons had completed at least one follow-up evaluation (96.6% of 1722 eligible survivors), with a mean of 7.5 completed annual evaluations per participant (SD = 3.9; range: 2–20). To date, 610 persons have developed incident MCI and 391 have developed incident dementia. There have been 788 deaths. Of these, 661 have had a brain autopsy (83.9%) followed by a uniform neuropathologic examination.

In summary, the Rush Memory and Aging Project includes all of the design and operational features that are standard for an analytic epidemiologic cohort study of incident disease, and nested within the study is organ donation. This provides a useful platform from which to investigate the neurobiological mechanisms linking psychosocial behavior with cognitive functioning in old age. Although participants are not enrolled in early life, they all undergo a life course interview and agree to organ autopsy and detailed neuropathological examination at death. Importantly, this allows for testing whether risk factors are related to cognition/dementia through a direct association with dementia-related pathologies, as might be expected for a traditional risk factor or in the case of reverse causation; by somehow modifying the association of neuropathological burden with cognition; or by some mechanism that is independent of the pathologies traditionally linked with cognitive aging and dementia. Details of all variables obtained in the study can be found at [129].

### 3.2. Psychosocial Research

The Rush Memory and Aging Project assesses a range of psychosocial behaviors and many of these behavioral measures have been associated with the subsequent rate of cognitive decline, risk of incident cognitive diagnoses (i.e., MCI, dementia), or both. These include anxiety-related traits (i.e., neuroticism [130,131], especially anxiety and vulnerability facets [132], and harm avoidance [133]), conscientiousness [134], state measures of psychological distress (i.e., depressive symptoms [135]), social [136,137] and emotional [136] isolation, and purpose in life [138].

Despite these links between psychosocial behavior and late-life cognitive health, for the most part, psychosocial measures have not been correlated with neuropathologic changes traditionally associated with dementia in old age. Exceptions are that a higher level of the harm avoidance trait [139] and lower level of the well-being dimension of purpose in life [140] have been associated with a higher likelihood of cerebral infarction. However, infarcts appear to account for a relatively small proportion of late-life cognitive variability [18,19], making it unlikely that infarcts mediate much of the association of these measures with cognition. It is notable that these clinical-pathologic studies have not found evidence that psychosocial measures (including neuroticism [130], harm avoidance [133], depressive symptoms [133], depressive disorder [141], loneliness [136], and purpose in life [142]) are related to common markers of neurodegeneration, including neuritic plaques, neurofibrillary tangles, Lewy bodies, TDP-43, and hippocampal sclerosis. These data do not support the reverse causality hypothesis. That is, it does not appear that psychosocial measures predict dementia because they are early signs of the disorder. This conclusion is also consistent with evidence from the Rush Memory and Aging Project and the Religious Orders Study that there is not an uptick in depressive symptoms preceding the diagnosis of dementia [133,143].

If psychosocial behavior is not strongly associated with dementia-related pathology, why is it related to dementia? One possibility is that it may somehow modify the deleterious association of pathology with cognition. Thus, in one report, the association of the neurofibrillary tangle density with a lower level of cognition proximate to death was reduced in individuals with larger social networks compared to those with smaller networks [144]. In a later report, the association of tangles with both a level of cognitive function and rate of cognitive decline proximate to death was reduced in persons with high levels of purpose in life compared to those with low levels of purpose [142]. These results suggest that the negative impact of an important component of Alzheimer’s disease pathology on cognition may be partly conditional on aspects of psychosocial behavior.

How psychosocial behavior might modify the brain’s response to gradual neurodegenerative changes is uncertain. One possible mechanism is through gene expression. Loneliness has been associated with the expression of inflammatory genes in peripheral leukocytes [97]. In a recent report, loneliness was associated with differential transcriptome expression in the nucleus accumbens, suggesting gene networks and regulatory mechanisms that might play a role in the association of loneliness with late-life cognitive health [145].

In addition to neuropathologies, we are also investigating the role of molecular and structural components of the neural elements that subserve cognition. For example, we found that select presynaptic proteins [18,146], neural density in the locus ceruleous [147], and the expression of brain derived neurotrophic factor [148] were all related to cognition. In one study, we found that depressive symptoms, purpose in life, and social isolation were related to cognitive decline, controlling for both neuropathologies and neuronal density in the locus [149]. These data suggest that the locus neuron density is not responsible for the effects of these psychosocial factors.

### 3.3. Future Directions

Current evidence supports the hypothesis that psychological and social experiences contribute to cognitive health in old age, but the underlying mechanisms remain uncertain. Although psychosocial factors are directly related to some neuropathologic conditions, it seems more likely that psychosocial factors work by contributing to changes in brain structure and function that modify how dementia-related pathologies are clinically expressed. By incorporating organ donation into an analytic epidemiologic cohort study, the study design of the Rush Memory and Aging Project offers unique opportunities to interrogate a wide range of molecular factors as potential mediators. Over the past decade, we have generated a wide range of molecular data including genome-, epigenome-, and transcriptome- wide data, and are now generating proteomics and metabolomics data [150]. In the future, these data, along with ante- and post-mortem neuroimaging [151], can be used to explore biologic mechanisms linking psychosocial risk factors to cognitive decline, MCI, and dementia. It is possible that common mechanisms will be found that underlie these associations and some of these mechanisms may serve as therapeutic targets offering a means of slowing cognitive decline agnostic to brain pathologies [152].
